# The Amylolytic Regulator AmyR of *Aspergillus niger* Is Involved in Sucrose and Inulin Utilization in a Culture-Condition-Dependent Manner

**DOI:** 10.3390/jof9040438

**Published:** 2023-04-04

**Authors:** Roland S. Kun, Sonia Salazar-Cerezo, Mao Peng, Yu Zhang, Emily Savage, Anna Lipzen, Vivian Ng, Igor V. Grigoriev, Ronald P. de Vries, Sandra Garrigues

**Affiliations:** 1 Fungal Physiology, Westerdijk Fungal Biodiversity Institute & Fungal Molecular Physiology, Utrecht University, Uppsalalaan 8, 3584 CT Utrecht, The Netherlands; 2 USA Department of Energy Joint Genome Institute, Lawrence Berkeley National Laboratory, 1 Cyclotron Rd., Berkeley, CA 94720, USA; 3 Department of Plant and Microbial Biology, University of California Berkeley, Berkeley, CA 94720, USA

**Keywords:** *Aspergillus niger*, transcription factors, AmyR, InuR, liquid cultivation, solid cultivation

## Abstract

Filamentous fungi degrade complex plant material to its monomeric building blocks, which have many biotechnological applications. Transcription factors play a key role in plant biomass degradation, but little is known about their interactions in the regulation of polysaccharide degradation. Here, we deepened the knowledge about the storage polysaccharide regulators AmyR and InuR in *Aspergillus niger*. AmyR controls starch degradation, while InuR is involved in sucrose and inulin utilization. In our study, the phenotypes of *A. niger* parental, Δ*amyR*, Δ*inuR* and Δ*amyR*Δ*inuR* strains were assessed in both solid and liquid media containing sucrose or inulin as carbon source to evaluate the roles of AmyR and InuR and the effect of culture conditions on their functions. In correlation with previous studies, our data showed that AmyR has a minor contribution to sucrose and inulin utilization when InuR is active. In contrast, growth profiles and transcriptomic data showed that the deletion of *amyR* in the Δ*inuR* background strain resulted in more pronounced growth reduction on both substrates, mainly evidenced by data originating from solid cultures. Overall, our results show that submerged cultures do not always reflect the role of transcription factors in the natural growth condition, which is better represented on solid substrates. Importance: The type of growth has critical implications in enzyme production by filamentous fungi, a process that is controlled by transcription factors. Submerged cultures are the preferred setups in laboratory and industry and are often used for studying the physiology of fungi. In this study, we showed that the genetic response of *A. niger* to starch and inulin was highly affected by the culture condition, since the transcriptomic response obtained in a liquid environment did not fully match the behavior of the fungus in a solid environment. These results have direct implications in enzyme production and would help industry choose the best approaches to produce specific CAZymes for industrial purposes.

## 1. Introduction

Plant biomass is the most abundant carbon source on Earth and is mainly composed of polysaccharides (cellulose, hemicellulose and pectin), the aromatic compound lignin, and storage polymers, such as starch and inulin [1,2]. Filamentous fungi secrete a wide arsenal of hydrolytic and oxidative enzymes that degrade these complex plant materials, and many of these enzymes have been commercially used for several industrial applications, such as pulp and paper, food and feed, detergents, textiles, and biofuel and biochemicals [3,4].

Starch degradation is performed by the production of amylolytic enzymes, (e.g., α-amylases, glucoamylases and α-glucosidases) which are mainly classified into the families of glycoside hydrolases GH13, GH15 and GH31 (http://www.cazy.org) [5,6]. Starch degradation is regulated at the transcriptional level in *Aspergillus* mainly by the transcriptional activator AmyR [7,8], which was the first GAL4-like transcription factor (TF) identified in filamentous fungi [9]. The role of AmyR in starch degradation has been studied in many *Aspergillus* species [10,11,12], although a broader role of this regulator was observed in *Aspergillus niger* [8,13,14,15]. AmyR triggers the production of amylolytic enzymes due to the presence of starch, maltose or low levels of D-glucose, which act as inducing compounds [8,16,17].

Inulin is another reserve carbohydrate found in plants, especially chicory, dahlia and Jerusalem artichoke [18]. Inulin-acting enzymes, such as exo-inulinases, inulin lyases and invertases, are fructofuranosyl hydrolases that target the β-2,1 linkages of inulin and hydrolyze it into fructose and glucose [19], and are an important class of industrial enzymes belonging to family GH32. The production of these enzymes is induced in the presence of inulin or sucrose, and has been reported to be under the control of a complex regulatory system [20,21], where the TF InuR plays the most dominant role. InuR acts as a positive transcriptional activator for the expression of genes involved in the breakdown of inulin and sucrose and the uptake of inulin-derived compounds, and has been reported to be closely related to AmyR [22]. These two regulators share structural similarities and putative DNA binding sites (CGGN_8_[C/A]GG) [12,22,23], indicating that they could have originated from the same ancestor gene. Despite these similarities, gene co-regulation or possible interaction of AmyR and InuR in the regulation of starch or inulin degradation has not been assessed yet in filamentous fungi.

In this study, we evaluated the influence of the amylolytic TF AmyR on the utilization of sucrose and inulin in the industrially relevant fungus *A. niger*. For this, single Δ*amyR* and Δ*inuR* deletion mutants, and the double Δ*amyR*Δ*inuR* deletion mutant were obtained using CRISPR/Cas9 genome editing technology [24], and phenotypical and transcriptomic analyses were performed. As we previously showed that different cultivation methods involving the use of solid or liquid medium highly affects the expression of genes involved in substrate degradation [25], we assessed the genetic response of the analyzed strains on both solid and liquid media containing sucrose or inulin as carbon sources. Results showed that AmyR contributes to the utilization of sucrose and inulin in *A. niger* together with InuR, which is represented by the further reduced growth and expression levels of Carbohydrate Active enZyme (CAZyme)-encoding genes in the Δ*amyR*Δ*inuR* strain compared to the single Δ*inuR* mutant when the fungus was grown on solid substrates compared to liquid.

## 2. Material and Methods

### 2.1. Strains, Media and Growth Conditions

*Escherichia coli* DH5α was grown in Luria–Bertani (LB) medium supplemented with 50 μg/mL ampicillin (Sigma-Aldrich, St. Louis, MO, USA) and was used for plasmid propagation. *A. niger* CBS 138852 [26] was used as a parental strain for construction of the mutants. The generated *A. niger* ∆*amyR*, ∆*inuR* and ∆*amyR*∆*inuR* mutants were deposited at the culture collection of Westerdijk Fungal Biodiversity Institute and the accession numbers are shown in Supplementary Table S1.

For strain propagation, *A. niger* control (CBS 138852), ∆*amyR*, ∆*inuR* and ∆*amyR*∆*inuR* strains were cultured in *Aspergillus* minimal medium (MM) or complete medium (CM) [27] at 30 °C supplemented with 1% D-glucose and 1.22 g/L uridine (Sigma-Aldrich). Conidia were harvested and dispersed in N-(2-acetamido)-2-aminoethanesulfonic acid (ACES) buffer, and their concentration was adjusted using a hemocytometer.

Growth profiles were carried out using MM containing 25 mM D-glucose, D-fructose, sucrose or maltose (Sigma-Aldrich), and 1% soluble starch (Difco^TM^, Wayne, NJ, USA) or inulin from chicory (Sigma-Aldrich). All media were supplemented with 1.22 g/L uridine. Plates were inoculated in duplicates with 2 µL containing 10^3^ conidia and incubated at 30 °C for up to 8 days. Plates were evaluated daily by visual inspection, taking into account colony diameter, mycelial density and sporulation. Pictures were taken after 8 days of incubation.

### 2.2. DNA Construction and Fungal Transformation

The ANEp8-Cas9-*pyrG* plasmid, which contains the autonomous fungal replicating element AMA1, *pyrG* as selection marker, *cas9* gene and the single guide RNA (sgRNA) expression construct under the control of the proline transfer ribonucleic acid (tRNA^Pro1^) promoter, was used in this study for the generation of fungal transformants [24]. The design of the 20 bp protospacers for the sgRNAs were performed using the Geneious 11.04.4 software tool (https://www.geneious.com). The sgRNA sequences (Supplementary Table S2) with no predicted off-targets and the highest on-target activity were designed based on the experimentally determined predictive model described by Doench et al. [28]. The sgRNAs for each gene were obtained to delete *amyR* (gene ID: NRRL3_07701) and *inuR* (gene ID: NRRL3_03593) genes in *A. niger* CBS 138852. All repair templates (RTs), which include the 5′ and 3′ flanking regions of the target genes, were obtained by fusion-PCR using Phusion High-Fidelity DNA Polymerase (Thermo Fisher Scientific, Waltham, MA, USA). Two PCR fragments were generated by amplifying approx. 750 bp upstream and downstream of the *inuR* and *amyR* genes. These two fragments were fused together in a second nested PCR obtaining ~1200 bp RT and were subsequently purified (Wizard^®^ SV Gel, Renton, WA, USA and PCR Clean-Up System, Promega, Madison, WI, USA).

**Figure 1:**
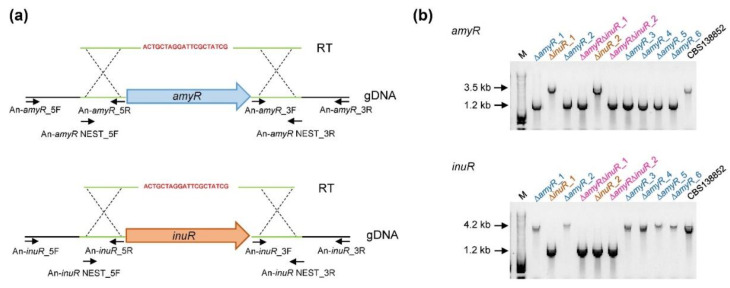
Molecular characterization of *A. niger* ∆*amyR*, ∆*inuR* and ∆*amyR*∆*inuR* strains. (**a**) Schematic representation of the genomic DNA (gDNA) reparation by homologous recombination after Cas9 cleavage in the *amyR* (blue arrow) and *inuR* (orange arrow) genes. The ~600 bp complementary arms to the upstream and downstream regions of *amyR* and *inuR* are represented in green. To generate the repair template (RT), a 20-bp sequence (red) was introduced for complementarity during fusion PCR. Black arrows represent the primers used for RT generation and transformant confirmation by PCR (Supplementary Table S2). Figure is not drawn to scale. (**b**) PCR confirmation of the *A. niger* ∆*amyR*, ∆*inuR* and ∆*amyR*∆*inuR* strains. Low bands (~1.2 kb) correspond to the deletants in *amyR* (top panel) and *inuR* (bottom panel), while upper bands (~3.5 and 4.2 kb) correspond to the parental strain (CBS 138852). Primer pairs used are located as indicated in the figure.

CRISPR/Cas9 plasmid construction, generation of *A. niger* protoplasts, transformation and purification of putative mutant strains were performed as previously described [29] with minor modifications [30]. Mutant strains were confirmed by PCR through the amplification of target gene region (Figure 1). All primers used in this study are shown in Supplementary Table S2 and were ordered from Integrated DNA Technologies (IDT, Leuven, Belgium).

### 2.3. Mycelial Dry Weight Measurement

For mycelial dry weight measurements from liquid cultures, 10^6^ conidia/mL of *A. niger* control, ∆*amyR*, ∆*inuR* and ∆*amyR*∆*inuR* strains were inoculated in 50 mL MM containing 1% sucrose or 1% inulin supplemented with 1.22 g/L uridine and 5 mM D-fructose to facilitate fungal germination. Triplicate samples were harvested after 48 h of cultivation in a rotary shaker at 250 rpm and 30 °C. For mycelial dry weight measurements from solid cultures, 10^3^ conidia of *A. niger* control, ∆*amyR*, ∆*inuR* and ∆*amyR*∆*inuR* strains were inoculated between two sterile polycarbonate track etched (PCTE) membrane layers (disc diameter 76 mm, PCTE 0.1 µm, Poretics^TM^, GVS Filter Technology, Zola Predosa, Italy) on MM plates containing 1% inulin or 1% sucrose as carbon source supplemented with 1.22 g/L uridine and 5 mM D-fructose. Non-sporulated mycelia were collected after 7 days of growth at 30 °C. Plates were inoculated in technical duplicates. Mycelia obtained from both liquid and solid cultures were subsequently dried o/n at 60 °C and weighed. Statistical significance was determined using ANOVA and Tukey HSD test. Significance was regarded as *p* < 0.05.

### 2.4. SDS-PAGE Assays

For protein production analysis, 10^6^ spores/mL of *A. niger* control, ∆*amyR*, ∆*inuR* and ∆*amyR*∆*inuR* mutant strains were pre-cultured in 250 mL CM containing 2% D-xylose and 1.22 g/L uridine for 16 h at 30 °C and 250 rpm. Mycelia (~2.5 g wet weight) were transferred to 50 mL MM containing 1% inulin or 1% soluble starch as carbon source, supplemented with 1.22 g/L uridine, and were incubated at 30°C and 250 rpm. Supernatant samples were taken after 4, 8, 24 and 32 h of incubation and were centrifuged for 10 min at 13,500× *g*. Ten μL of each sample were analyzed by SDS-PAGE using SDS-12% polyacrylamide gels calibrated with PageRuler™ Plus Prestained Protein Ladder (Thermo Scientific) and silver stained [31]. Samples were evaluated in biological triplicates.

### 2.5. Transcriptomics Analysis

For transcriptomics analysis of liquid culture samples, freshly harvested spores from *A. niger* control, ∆*amyR*, ∆*inuR* and ∆*amyR*∆*inuR* strains were pre-grown in 250 mL CM containing 2% D-xylose and 1.22 g/L uridine for 16 h at 30 °C in a rotary shaker at 250 rpm. After 16 h, mycelia were harvested by filtration through sterile cheesecloth, rinsed with MM, and approximately 2.5 g (wet weight) mycelium was transferred into 50 mL MM containing 1% sucrose, and 1% inulin supplemented with 1.22 g/L uridine. Mycelia were collected after 2 and 8 h and were frozen in liquid nitrogen followed by storage at −80 °C. Samples were collected in triplicates.

For transcriptomics analysis of solid culture samples, 10^3^ conidia of *A. niger* control, ∆*amyR*, ∆*inuR* and ∆*amyR*∆*inuR* strains were inoculated and pre-grown between two sterile polycarbonate track etched (PCTE) membrane layers (disc diameter 76 mm, PCTE 0.1 µm, Poretics^TM^, GVS Filter Technology) on MM plates containing 1% soybean hulls as carbon source supplemented with 1.22 g/L uridine at 30 °C. After two days of pre-culturing, the polycarbonate membrane layers containing the fungal mycelia were transferred to MM plates containing 1% sucrose or 1% inulin supplemented with 1.22 g/L uridine and were grown at 30 °C. Mycelia were collected after 40 h of growth and were frozen in liquid nitrogen and stored at −80 °C. Samples were collected in triplicates.

The transcriptomes of the control and deletion mutant strains cultivated for 2 and 8 h in liquid cultures and cultivated for 40 h on solid cultures were analyzed using RNA-seq. RNA isolation, purification, and quantitative and qualitative evaluation were performed as previously described [32].

For liquid culture samples, purification of mRNA, synthesis of cDNA library and sequencing were performed at the Joint Genome Institute (JGI, US). RNA sequencing, processing of raw fastq reads and evaluation of raw gene counts were performed as previously reported [32]. Three biological replicates were generated and sequenced for each condition. Two individual samples were discarded from further analysis due to their poor sequencing quality.

For solid culture samples, the preparation of libraries and sequencing of the mRNA along with the analysis of the raw data were performed by GenomeScan B.V. The RNA concentration and integrity were assessed using the Agilent Fragment Analyzer system. Subsequently, samples were processed using the NEBNext Ultra II directional RNA library prep kit for Illumina^®^. Briefly, rRNA was depleted from total RNA using the rRNA depletion kit (Qiagen fast select). After fragmentation of the rRNA-reduced RNA, a cDNA synthesis was performed. This was used for ligation with the sequencing adapters and PCR amplification of the resulting product. The quality and yield after sample preparation was measured with the fragment analyzer. The size of the resulting products was consistent with the expected size distribution (a broad peak between 300 and 500 bp). Clustering and DNA sequencing were performed using Illumina Novaseq6000 in line with manufacturer’s instructions at the concentration of 1.1 nM of DNA. Image analysis, base calling and the quality check were conducted using the Illumina data analysis pipeline RTAv3.4.4 and Bclfastqv2.20. Data obtained from the Novaseq6000 in fastq format was used as source for the downstream data analysis. Alignment of fastq reads was performed using STAR2 version v2.5.4b against *A. niger* NRRL3. The aligned reads were stored in a sorted BAM format and indexed using Samtools v1.95. For the removal of PCR duplicates within the library Picard MarkDuplicates v2.23.66 was used. The deduplicated mapped reads are counted for each exonic feature in the reference GTF annotation using htseq-count v0.11.07.

In all cases, differentially expressed genes (DEGs) were detected using the R package DESeq2 [33]. Transcripts were considered differentially expressed if the DESeq2 fold change of the deletion mutant strains compared to the control was >2 (upregulation) or <0.5 (downregulation) and *p*adj < 0.01 (the false discovery rate (FDR)-corrected *p*-value derived from default DESeq2 output based on the Benjamini–Hochberg procedure) and at least one of the two expression values was FPKM > 20.

## 3. Results and Discussion

### 3.1. Generation of ∆amyR, ∆inuR and ∆amyR∆inuR Strains in A. niger in a Single Transformation Event

In order to obtain *A. niger* ∆*amyR*, ∆*inuR* and ∆*amyR*∆*inuR* strains, one single transformation event was performed in which 1 µg of each ANEp8-Cas9-*pyrG* plasmid carrying sgRNAs targeting *amyR* or *inuR* were mixed and co-transformed together with 5 µg of each corresponding RT into *A. niger* protoplasts as previously described [30]. Forty-eight transformants were obtained and ten were further isolated to monosporic culture and screened. From these ten, six transformant colonies were positive ∆a*myR* mutants, two were positive ∆*inuR* mutants and two were double ∆*amyR*∆*inuR* mutants (Figure 1). All single and combinatorial mutants were identified by PCR using specific primers designed to differentiate between mutants and the control strain (Supplementary Table S2) (Figure 1).

### 3.2. The Amylolytic Transcription Factor AmyR Influences Growth of A. niger on Sucrose and Inulin

**Figure 2:**
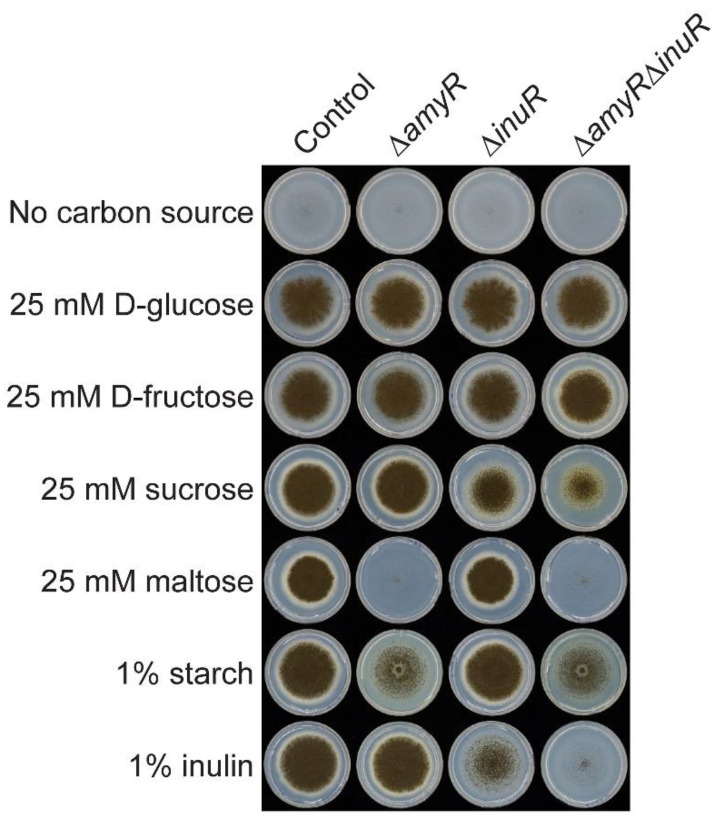
Growth profile of *A. niger* control, ∆*amyR*, ∆*inuR* and ∆*amyR*∆*inuR* strains grown on starch, inulin and other starch/inulin-related carbon sources after 8 days of incubation at 30 °C.

To evaluate the contribution of the amylolytic and inulinolytic transcriptional activators AmyR and InuR, respectively, to the utilization of storage polysaccharides and related carbon sources in *A. niger*, the parental, ∆*amyR*, ∆*inuR* and double ∆*amyR*∆*inuR* strains were grown on agar plates containing different carbon sources. These include D-glucose, D-fructose, sucrose, maltose, starch and inulin (Figure 2). Growth on D-glucose and D-fructose was similar for all strains, whereas ∆*inuR* showed reduced growth on sucrose and inulin compared to the control and ∆*amyR* strains, as previously reported [22]. These results confirm the involvement of InuR in sucrose and inulin utilization in *A. niger*. The ∆*amyR*∆*inuR* double deletion strain showed a further reduced growth on both sucrose and inulin compared to the single ∆*inuR* strain*,* suggesting a role for AmyR in sucrose and inulin utilization in *A. niger*. Finally, the growth on maltose and starch was only affected by the deletion of *amyR*, confirming the dominant role of AmyR in starch utilization [10,11]. 

### 3.3. The Contribution of AmyR to Sucrose and Inulin Utilization Depends on the Cultivation Method

**Figure 3:**
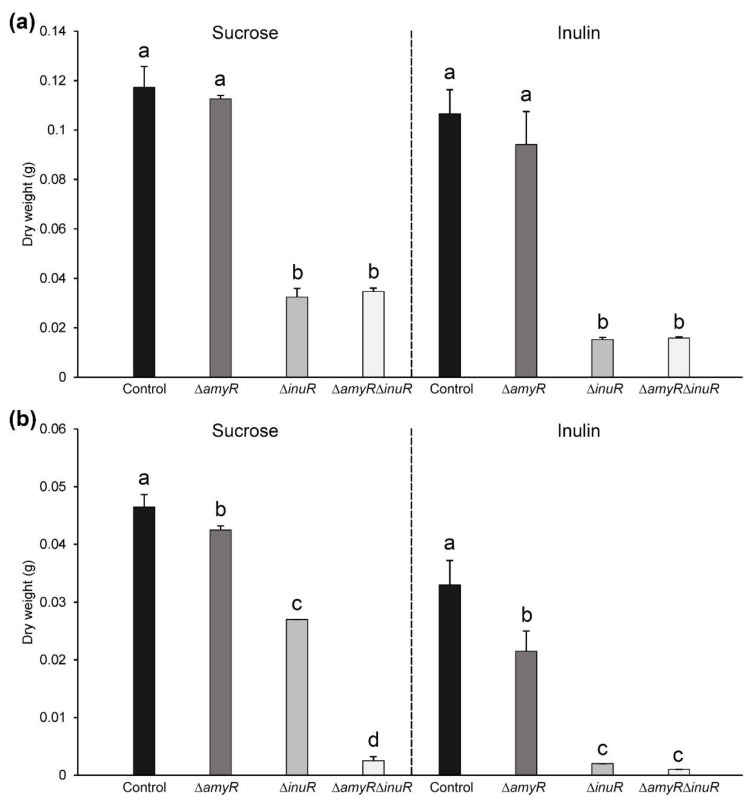
Mycelial dry weight measurement results of *A. niger* control (CBS 138852), ∆*amyR*, ∆*inuR* and ∆*amyR*∆*inuR* strains grown on liquid (**a**) or solid (**b**) medium containing 1% sucrose or inulin as carbon source, supplemented with 1.22 g/L uridine and 5 mM D-fructose to facilitate initial germination of fungal conidia. Liquid culture triplicates were incubated for 48 h in a rotary shaker at 250 rpm and 30 °C, while solid medium cultures were grown for 7 days at 30 °C. Samples showing different letters (a–d) show statistically significant differences among the strains (ANOVA and Tukey HSD test, *p* < 0.05).

In order to evaluate the influence of AmyR on growth on sucrose and inulin, the mycelial weight of *A. niger* control, ∆*amyR*, ∆*inuR* and double ∆*amyR*∆*inuR* strains was evaluated from both liquid and solid medium cultures (Figure 3). In both cases, the media contained either 1% sucrose or 1% inulin as carbon source, supplemented with 5 mM D-fructose to facilitate initial germination of fungal spores. In case of liquid cultures, the deletion of *inuR* resulted in substantially decreased mycelial weight in liquid minimal medium (MM) containing 1% sucrose or inulin (average weight of 32 mg and 15 mg, respectively) compared to the control (117 mg and 107 mg, respectively) after 48 h of growth (*p <* 0.05) (Figure 3a). However, dry weight measurement results from liquid cultures did not suggest the contribution of AmyR to growth on sucrose or inulin, indicated by the comparable growth of Δ*amyR* and Δ*amyR*Δ*inuR* strains to that of the control and Δ*inuR* strain, respectively (Figure 3a). SDS-PAGE analysis showed reduced protein production by the Δ*amyR*Δ*inuR* mutant compared to the Δ*inuR* strain after 24 h and 32 h of growth (Supplementary Figure S1). However, the mycelial weight measurement results indicate that most proteins abolished by the additional deletion of *amyR* in the Δ*inuR* strain are probably not essential for inulin utilization.

In contrast to the dry weight measurements from liquid cultures, when the tested strains were cultivated on solid MM plates containing the same carbon sources, the Δ*amyR* strain showed reduced mycelial weight on both substrates compared to the control (Figure 3b). Moreover, the Δ*amyR*Δ*inuR* double deletion strain showed significantly reduced mycelial weight on sucrose compared to the Δ*inuR* deletion strain (2.5 mg compared to 27 mg) (*p <* 0.05), which correlates with our growth profile results (Figure 2). Despite the reduced mycelial weight of the Δ*amyR* strain compared to the control on inulin (Figure 3b) and the reduced growth of Δ*amyR*Δ*inuR* mutant compared to the Δ*inuR* strain on the same substrate (Figure 2), the mycelial weight of Δ*inuR* and Δ*amyR*Δ*inuR* was comparable on inulin in both culture conditions (Figure 3).

Overall, these results indicate that AmyR influences the growth on sucrose, and to a lesser extent on inulin, but only when the fungus is cultivated on solid medium.

### 3.4. A. niger AmyR Shows Minor Involvement in the Regulation of Gene Expression When the Fungus Is Cultivated in Liquid Medium Containing Sucrose and Inulin

In order to evaluate the regulatory mechanisms playing a role in sucrose and inulin utilization on a molecular level, transcriptome data were generated from liquid cultures of *A. niger* control, Δ*amyR*, Δ*inuR* and Δ*amyR*Δ*inuR* strains. Samples were taken after 2 and 8 h of growth following a transfer from CM medium containing 2% D-xylose to MM medium containing 1% sucrose or 1% inulin.

**Figure 4:**
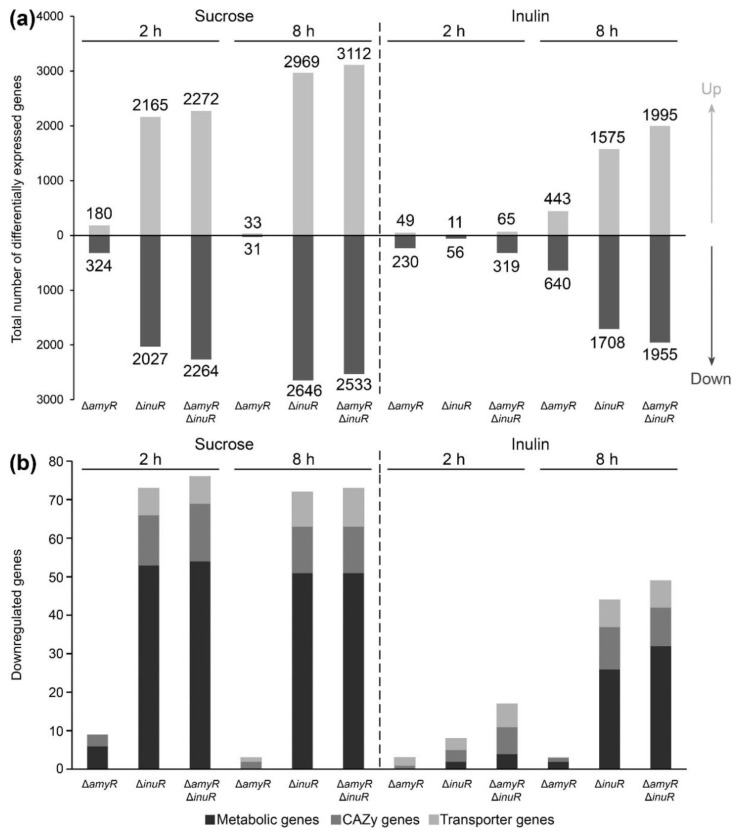
Differentially expressed genes (DEGs) in *A. niger* Δ*amyR*, Δ*inuR* and Δ*amyR*Δ*inuR* liquid cultures compared to the control (CBS 138852). Samples originated from 2 or 8 h of growth in liquid minimal medium containing 1% sucrose or 1% inulin as carbon source. (**a**) Total number of DEGs across the genome. Up- or downregulated genes are indicated by different tints of grey. (**b**) Downregulated plant biomass utilization-related genes. CAZy, metabolic and transporter genes are indicated by different tints of grey.

Considering the genome-wide response of the fungus, the deletion of either *amyR*, *inuR* or both resulted in relatively low number of DEGs compared to the control on inulin after 2 h of growth (Figure 4a, right panel). However, after 8 h of growth, the double deletion of *amyR* and *inuR* resulted in the upregulation of 1995 and downregulation of 1955 genes, which can mostly be associated with the deletion of *inuR*, showing the upregulation and downregulation of 1575 and 1708 genes, respectively (Figure 4a, right panel). In contrast to the inulin culture, the number of DEGs was substantially higher at both timepoints when grown on medium containing 1% sucrose as carbon source, mainly affected by the deletion of *inuR* (Figure 4a, left panel). Interestingly, the Δ*inuR* strain showed a higher number of downregulated genes compared to the Δ*amyR*Δ*inuR* strain after 8 h of cultivation on sucrose, highlighting the major role of InuR for the utilization of this substrate (Figure 4a, left panel). The large number of upregulated genes in the *inuR* single and double deletion mutants correlates with the upregulation of major regulators involved in plant biomass degradation (Supplementary Figure S2). The upregulation of major TF genes might be an indirect consequence of the downregulation of *creA*, resulting in a carbon catabolite de-repressed phenotype [34] (Supplementary Figure S2). The relation of AmyR and InuR to the utilization of sucrose and inulin on a genetic level correlates with the mycelial weight results originating from liquid cultures (Figure 3a).

The expression level of a set of 481 plant biomass utilization-related genes, including 217 CAZy, 168 metabolic genes, 85 transporter genes and 11 TF genes, were analyzed to evaluate the involvement of both TFs in the regulation of sucrose and inulin utilization in more detail. As expected, the deletion of *inuR* showed the highest individual impact on CAZy, metabolic and transporter gene expression when grown on the test substrates (Figure 4b, Supplementary Data S1). A large number of plant biomass utilization-related genes were downregulated in the Δ*inuR* mutant at both timepoints on sucrose (72 and 71 genes after 2 and 8 h of growth, respectively), while only 8 and 46 genes were downregulated on inulin after 2 and 8 h of growth, respectively (Figure 4b). Therefore, only a small set of 7 genes were affected by the deletion of *inuR* in all four experimental conditions (Table 1). These include *sucA/suc1*, *inuE/inu1*, *mstH* and the putative maltose/sucrose transporter gene NRRL3_3594 [35]. Based on our results, an additional putative inositol/fructose transporter gene, NRRL3_11807 [35], is controlled by InuR. The expression of *aglC* was substantially reduced in the Δ*inuR* strain compared to the control. Similarly, reduced expression of *aglC* was observed in a Δ*amyR* mutant when grown on maltose and starch, but not on D-glucose [8], which was shown to induce the expression of *aglC* [36]. Therefore, in our study, the reduced expression of *aglC* in the Δ*inuR* strain is most likely related to impaired release of D-glucose from sucrose and inulin. However, the dependency of *aglC* expression on D-glucose concentration is not fully understood. The previously reported InuR dependent genes, *inuA* and *sucB* [22], were also downregulated in the Δ*inuR* mutant. However, these genes showed generally low expression (FPKM < 20) in the control strain and were excluded from the analysis (Supplementary Data S1). Interestingly, the invertase gene, *sucC*, was not expressed in any of our test conditions (Supplementary Data S1).

In contrast, the deletion of *amyR* did not indicate a substantial contribution of AmyR to sucrose and inulin utilization when cultivated in liquid medium. Only three CAZy and six metabolic genes were downregulated in the Δ*amyR* strain compared to the control after 2 h of growth on sucrose, while an even lower number of genes were downregulated in any of the other conditions. Moreover, only a putative glycerol proton symporter gene (NRRL3_817) [35] showed significantly decreased expression in the Δ*amyR*Δ*inuR* strain compared to the Δ*inuR* single deletion mutant after 8 h of growth on inulin.

Overall, gene expression data generated from liquid cultures did not support an involvement of AmyR in sucrose or inulin utilization at the transcriptomic level, which correlates with the mycelial dry weight measurement results from liquid cultures (Figure 3a).

### 3.5. AmyR Contributes to the Utilization of Sucrose and Inulin When A. niger Is Grown on Solid Media

In order to explain the impact of *amyR* deletion on growth on solid medium containing 1% sucrose or 1% inulin as carbon source (Figure 2 and Figure 3b), transcriptomic data were generated from *A. niger* control, Δ*amyR,* Δ*inuR* and Δ*amyR*Δ*inuR* cultures grown on solid agar plates. 

**Figure 5:**
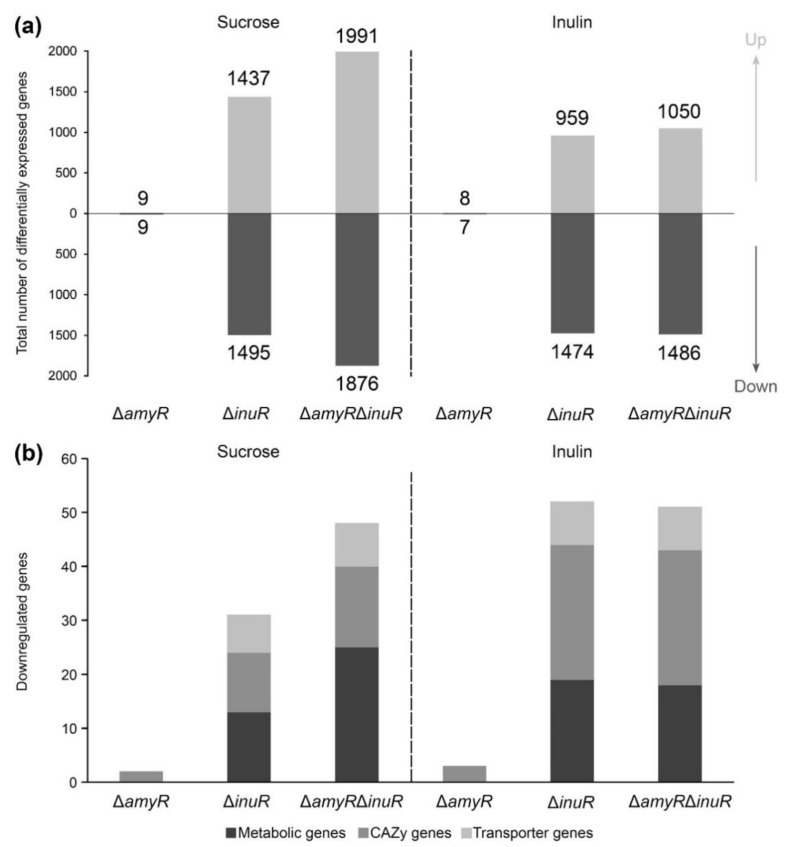
Differentially expressed genes (DEGs) in *A. niger* Δ*amyR*, Δ*inuR* and Δ*amyR*Δ*inuR* solid cultures compared to the control (CBS 138852). Samples originated from 40 h of growth on solid minimal medium containing 1% sucrose or 1% inulin as carbon source. (**a**) Total number of DEGs across the genome. Up- or downregulated genes are indicated by different tints of grey. (**b**) Downregulated plant biomass utilization-related genes. CAZy, metabolic and transporter genes are indicated by different tints of grey.

Genome-wide transcriptome results indicated that AmyR has a minimal impact on sucrose and inulin utilization in the presence of InuR. This was evidenced by the low number of DEGs in the Δ*amyR* strain compared to the control on both substrates (Figure 5a). However, the additional deletion of *amyR* in the Δ*inuR* background strain resulted in further upregulation and downregulation of 554 and 381 genes on sucrose, respectively (Figure 5a).

In total, 31 plant biomass utilization-related genes, including 13 CAZy, 14 metabolic and 4 transporter genes, were downregulated in the double deletion strain compared to Δ*inuR* on sucrose. In contrast, a very small number of plant biomass utilization-related genes showed downregulation in the double deletion strain compared to the Δ*inuR* on inulin (Figure 5b), which can be associated with the very low expression of *amyR* (3.83 FPKM) in the Δ*inuR* strain on inulin (Supplementary Data S2). Moreover, mycelial weight measurements did not indicate either significant difference between the Δ*inuR* and Δ*amyR*Δ*inuR* strains on inulin (Figure 3).

The involvement of AmyR in sucrose and inulin utilization is most likely mediated through the regulation of α-glucosidase activity. This activity has been reported to play a role in sucrose utilization [37,38,39] and can be involved in the removal of terminal α-D-linked glucose units from inulin. A previous study indicated that the expression of α-glucosidase-encoding genes *agdA* and *agdB* was reduced when *amyR* was deleted [22]. In contrast, our results showed increased expression of *agdB* in the Δ*amyR*Δ*inuR* strain compared to the control when grown on solid sucrose and inulin-containing medium (Supplementary Table S3). In fact, *agdB* was the highest expressed CAZy gene in the Δ*amyR*Δ*inuR* strain cultivated on both solid carbon sources (Supplementary Data S2), correlating with previous observations that the expression of this gene is not AmyR dependent [14]. However, the expression of *agdA* was significantly downregulated in both Δ*amyR* and Δ*amyR*Δ*inuR* deletion strains compared to the control when cultivated on solid medium containing sucrose or inulin as carbon source. Moreover, the deletion of *inuR* resulted in the upregulation of *agdA* compared to the control (302.13 FPKM compared to 68.86 FPKM, respectively) (Supplementary Table S3 and Supplementary Data S2) on solid medium containing sucrose, most likely to compensate for the reduced expression of invertase-encoding genes (eg., *sucA* and *sucB*) [40]. Therefore, the additional deletion of *amyR* in the Δ*inuR* strain and the subsequent downregulation of *agdA* (15.13 FPKM) (Supplementary Table S3 and Supplementary Data S2) could explain the significant growth reduction of the Δ*amyR*Δ*inuR* strain compared to Δ*inuR* on sucrose (Figure 2 and Figure 3b).

**Table 1:** Downregulated genes in the *A. niger* Δ*inuR* strain compared to the control in liquid media. The genes included in this table were downregulated in both media containing 1% sucrose or 1% inulin after 2 and 8 h of growth. Gene expression is represented as FPKM values.

Gene Number	Gene Name	Description	Reference	Sucrose				Inulin			
				Control 2 h	Δ*inuR* 2 h	Control 8 h	Δ*inuR* 8 h	Control 2 h	Δ*inuR* 2 h	Control 8 h	Δ*inuR* 8 h
NRRL3_11752	*sucA/suc1*	SUC (invertase/β-fructofuranosidase)	[41]	1606.86	0.25	218.88	2.79	220.34	0.16	312.10	0.48
NRRL3_11807	-	Putative inositol/fructose transporter	[35]	376.17	51.66	235.22	22.16	572.01	21.53	1474.03	29.23
NRRL3_16	*aglC*	AGL (α-1,4-galactosidase)	[36]	469.34	25.44	30.79	3.91	127.91	15.72	97.77	2.57
NRRL3_3087	*inuE/inu1*	INX (exo-inulinase)	[42]	1716.50	1.78	168.76	2.85	1352.05	1.41	1681.95	1.24
NRRL3_3594	-	Putative maltose/sucrose transporter	[35]	84.89	12.34	44.87	3.97	134.79	9.96	170.26	5.79
NRRL3_3879	*mstH*	High-affinity glucose transporter	[43]	662.64	60.42	579.77	17.08	385.58	49.97	1084.95	10.74
NRRL3_8073	*pycA*	Pyruvate carboxylase	[44]	5154.97	379.43	650.99	292.21	638.20	303.34	1705.41	524.39

## 4. Conclusions

In conclusion, data originating from liquid culture samples indicated no involvement of AmyR in sucrose and inulin utilization. However, growth profiling, analysis of mycelial dry weight and transcriptome data originated from solid medium cultures indicated substantial involvement of AmyR in the regulation of sucrose utilization, as well as in the regulation of inulin utilization to a limited extent. Our data showed that the amylolytic regulator AmyR is partially involved in the utilization of sucrose and inulin in *A. niger*, by controlling the expression of α-glucosidase genes. In particular, the expression of the AmyR-dependent gene *agdA* correlates with the ability of *A. niger* to grow on sucrose and inulin when the major inulinolytic regulator, InuR, is deleted. In contrast, *agdB* does not show an AmyR dependent expression, and the residual growth of Δ*amyR*Δ*inuR* strain observed on sucrose and inulin could be associated with the activity of AgdB.

Overall, these results show that submerged cultures, which are most often used in laboratory and industrial setups, do not always reflect the role of TFs in the natural growth conditions of the fungus, which is better represented on solid substrates. The phenotypical differences between the liquid and solid culture conditions observed in this study could be attributed to different environmental conditions, including aeration, oxygen diffusion rate, viscosity, osmolarity, agitation, cell density or substrate availability [25].

## Data Availability

The raw RNAseq data originating from liquid culture samples were deposited at the Sequence Read Archive at NCBI with sample accession numbers SRP379898-379907, SRP379909-379920 and SRP379922-379945. The raw RNAseq data originating from solid culture samples were deposited to GEO with accession ID GSE204768. All other data are available in the main text or in the supplementary files.

## Supplementary Materials

The following supporting information can be downloaded at: https://www.mdpi.com/article/10.3390/jof9040438/s1. Supplementary Figure S1. Protein production analysis of *A. niger* parental, ∆*amyR*, ∆*inuR* and ∆*amyR*∆*inuR* strains. Supplementary Figure S2. Hierarchical clustering of transcription factor genes in *A. niger* control (CBS 138852) and Δ*amyR*, Δ*inuR* and Δ*amyR*Δ*inuR* deletion mutants. Supplementary Table S1. *A. niger* strains used in this study. Supplementary Table S2. Primers used in this study. Supplementary Table S3. Expression levels of two major α-glucosidase genes, *agdA* and *agdB*, in the *A. niger* control (CBS 138852) and Δ*amyR*, Δ*inuR* and Δ*amyR*Δ*inuR* deletion strains cultivated on solid media containing 1% sucrose or inulin as carbon source. Supplementary Data S1. Gene expression values of CAZy-, metabolic-, sugar transporter- and transcription factor genes of *A. niger* control (CBS 138852) and deletion mutant strains cultivated in liquid media. Supplementary Data S2. Gene expression values of CAZy-, metabolic-, sugar transporter- and transcription factor genes of *A. niger* control (CBS 138852) and deletion mutant strains cultivated on solid media.
