# Supplementary material for: The Amylolytic Regulator AmyR of Aspergillus niger Is Involved in Sucrose and Inulin Utilization in a Culture-Condition-Dependent Manner
Source: J Fungi (Basel). 2023 Apr 4;9(4):438. doi: 10.3390/jof9040438 (PMC10142829; doi:10.3390/jof9040438)
Supplement: Supplementary file 1 [file jof-09-00438-s001.zip › Supplementary material.pdf]

Supplementary material

Title

The amylolytic regulator AmyR of *Aspergillus niger* is involved in sucrose and inulin utilization in a culture condition-dependent manner

Authors

Roland S. Kun [1], Sonia Salazar-Cerezo [1], Mao Peng [1], Yu Zhang [2], Emily Savage [2], Anna Lipzen [2], Vivian Ng [2], Igor V. Grigoriev [2,3], Ronald P. de Vries [1,*] and Sandra Garrigues [1,*,†]

Affiliations

[1] Fungal Physiology, Westerdijk Fungal Biodiversity Institute & Fungal Molecular Physiology, Utrecht University, Uppsalalaan 8, 3584 CT Utrecht, The Netherlands

[2] USA Department of Energy Joint Genome Institute, Lawrence Berkeley National Laboratory, 1 Cyclotron Rd, Berkeley, CA 94720, United States

[3] Department of Plant and Microbial Biology, University of California Berkeley, Berkeley, CA 94720, United States

[†] Current address: Food Biotechnology Department, Instituto de Agroquímica y Tecnología de Alimentos (IATA), Consejo Superior de Investigaciones Científicas (CSIC), Catedrático Agustín Escardino Benlloch 7, 46980 Paterna, Valencia, Spain

* Correspondence: Ronald P. de Vries: r.devries@wi.knaw.nl and Sandra Garrigues: sgarrigues@iata.csic.es

Table of contents

Supplementary Figure S1. Protein production analysis of *A. niger* parental, Δ*amyR*, Δ*inuR* and Δ*amyR*Δ*inuR* strains.

Supplementary Figure S2. Hierarchical clustering of transcription factor genes in *A. niger* control (CBS 138852) and Δ*amyR*, Δ*inuR* and Δ*amyR*Δ*inuR* deletion mutants.

Supplementary Table S1. *A. niger* strains used in this study.

Supplementary Table S2. Primers used in this study.

Supplementary Table S3. Expression levels of two major α-glucosidase genes, *agdA* and *agdB*, in the *A. niger* control (CBS 138852) and Δ*amyR*, Δ*inuR* and Δ*amyR*Δ*inuR* deletion strains cultivated on solid media containing 1% sucrose or inulin as carbon source.

Supplementary Figure S1: Protein production analysis of *A. niger* parental, Δ*amyR*, Δ*inuR* and Δ*amyR*Δ*inuR* strains. SDS-PAGE analysis of the cell-free supernatants after 4, 8, 24 and 32 h of growth on MM + 1% inulin. Ten µL of supernatant were loaded per well. Three biological samples are shown per strain. M: PageRuler™ Plus Prestained Protein Ladder (Thermo Scientific).

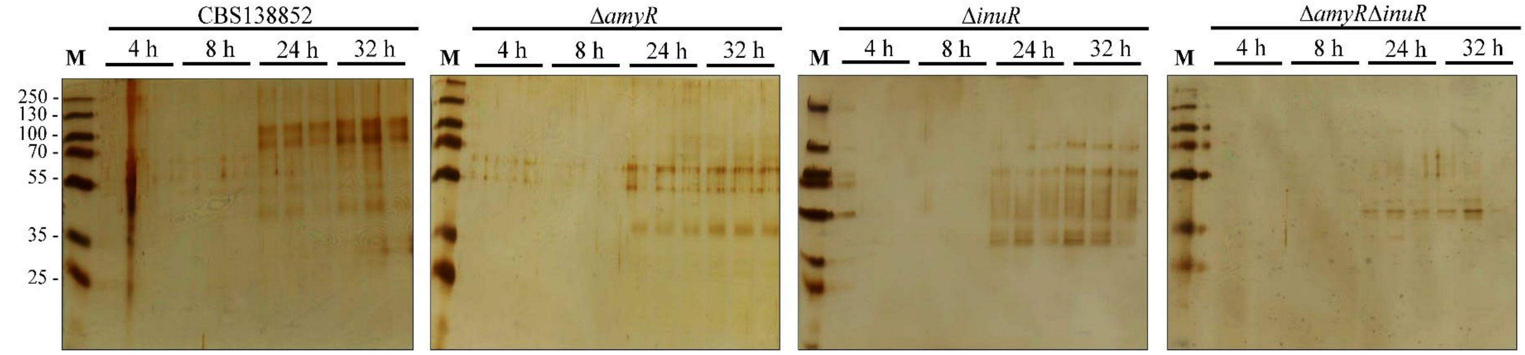

Supplementary Figure S2: Hierarchical clustering of transcription factor genes in *A. niger* control (CBS 138852) and Δ*amyR*, Δ*inuR* and Δ*amyR*Δ*inuR* deletion mutants. Data originated from 2 and 8 h of culturing in 1% sucrose (**A**) or 1% inulin (**B**) liquid minimal medium. Downregulated genes (fold change < 0.5; *p*adj < 0.01) in the deletion mutants compared to the control are indicated by a downward arrow. Upregulated genes (fold change > 2; *p*adj < 0.01) are indicated by an upward arrow. The analyzed genes include the genes encoding the carbon catabolite repressor CreA, the (hemi-)cellulolytic regulators ClrA and ClrB, the xylanolytic regulator XlnR, the arabinanolytic regulator AraR, the amylolytic regulator AmyR, the inulinolytic regulator InuR, the regulator of L-rhamnose utilization RhaR, the regulator of D-galactose utilization GalX and the activator and repressor of D-galacturonic acid utilization GaaR and GaaX, respectively.

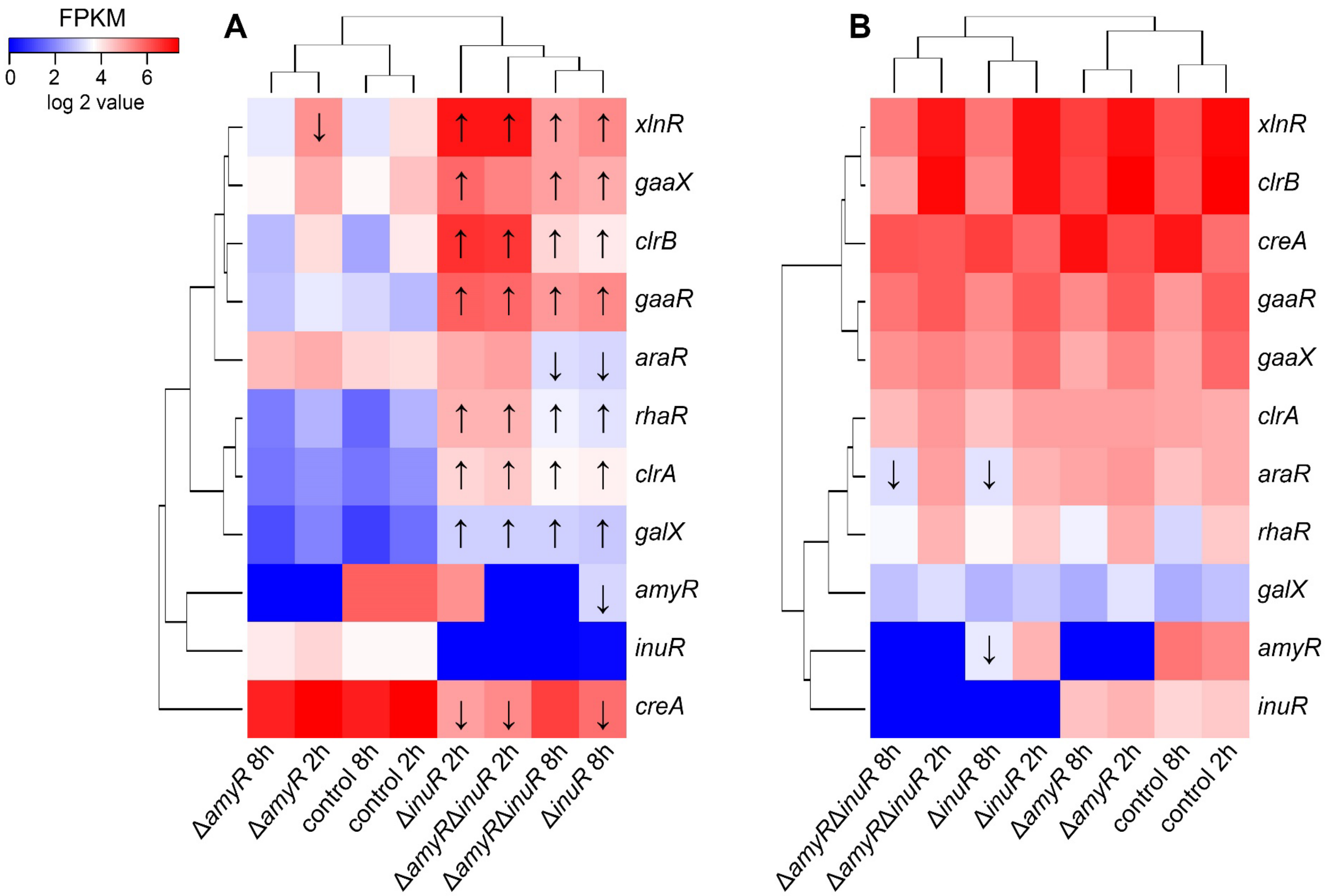

Supplementary Table S1. *A. niger* strains used in this study.

CBS number	Strain description	Genotype	Reference
CBS 138852	N593 Δ*kusA*	*cspA1*, *pyrG*⁻, *kusA::amdS*	[1]
CBS 146902	Δ*amyR*	*cspA1*, *pyrG*⁻, *kusA::amdS*, Δ*amyR*	This study
CBS 146903	Δ*inuR*	*cspA1*, *pyrG*⁻, *kusA::amdS*, Δ*inuR*	This study
CBS 146904	Δ*amyR* Δ*inuR*	*cspA1*, *pyrG*⁻, *kusA::amdS*, Δ*amyR*, Δ*inuR*	This study

[1] Meyer, V.; Arentshorst, M.; El-Ghezal, A.; Drews, A.C.; Kooistra, R.; van den Hondel, C.A.M.J.J.; Ram, A.F.J. Highly efficient gene targeting in the *Aspergillus niger kusA* mutant. *J. Biotechnol.* **2007**, 128, 770–775.

Supplementary Table S2. Primers used in this study**.**

Primer ID	Use[a]	Sequence (5'→3') [b]	Purpose[c]
P1	F	CAACCTCCAATCCAATTTGACTCCGCCGAACGTACTG	gRNA
P2	R	ACTACTCTACCACTATTTGAAAAGCAAAAAAGGAAGGTACAAAAAAGC	gRNA
P3-*amyR*	R	**TGCGATAAGTGTCGACGCCT**GACGAGCTTACTCGTTTCG	gRNA
P4-*amyR*	F	**AGGCGTCGACACTTATCGCA**GTTTTAGAGCTAGAAATAGCAAG	gRNA
P3-*inuR*	R	**ACGTCTTCTTCTCGAGCTAA**GACGAGCTTACTCGTTTCG	gRNA
P4-*inuR*	F	**TTAGCTCGAGAAGAAGACGT**GTTTTAGAGCTAGAAATAGCAAG	gRNA
Fw-screen	F	TTTTCTCTTCCATTTACGC	cse
Rev-screen	R	GGGGATCATAATAGTACTAGCCA	cse
An-*amyR*_5F	F	TGGGATGTTCACCAGTGTTACG	RT, csa
An-*amyR*_5R	R	CGATAGCGAATCCTAGCAGTGCGGAGACAAGTGTGACTCC	RT
An-*amyR*_3F	F	ACTGCTAGGATTCGCTATCGCAACTACGACGATGACGATGC	RT
An-*amyR*_3R	R	CACCGTGACCCAGAGAAAGG	RT, csa
An-*amyR* NEST_5F	F	GAGCCTCAGACTCTGTCAGC	RT
An-*amyR* NEST_3R	R	TCCACCACCATCAAAATCACC	RT
An-*inuR*_5F	F	TCGGAGGAGATACGCAAAGC	RT, csa
An-*inuR*_5R	R	CGATAGCGAATCCTAGCAGTCTCACGAGTCATAAGCATTGTCG	RT
An-*inuR*_3F	F	ACTGCTAGGATTCGCTATCGTGGCGTTCTGGTTTTATCACG	RT
An-*inuR*_3R	R	ATTGACGGGCATGAGTGTCC	RT, csa
An-*inuR* NEST_5F	F	TAAGAACAACGACCCCATCGC	RT
An-*inuR* NEST_3R	R	AAGGGAAACCGCCAATCAGC	RT

[a] F: forward; R: reverse.

[b] The gRNA sequence is highlighted in bold.

[c] gRNA: guide RNA; RT: Repair Template; cse: colony screening for *E. coli*; csa: colony screening for *A. niger*.

Supplementary Table S3. Expression levels of two major α-glucosidase genes, *agdA* and *agdB*, in the *A. niger* control (CBS 138852) and Δ*amyR*, Δ*inuR* and Δ*amyR*Δ*inuR* deletion strains cultivated on solid media containing 1% sucrose or inulin as carbon source. Gene expression values of FPKM < 20 were considered low, and are indicated in red. Upregulated genes (fold change > 2) are indicated in blue, while downregulated genes (fold change < 0.5) are indicated in orange. Statistically significant changes (*p* adj < 0.01) are indicated in bold.

Gene ID	Gene name	Gene expression values (FPKM)								DeSeq2 fold change compared to the control strain					
		Control sucrose	Δ*amyR* sucrose	Δ*inuR* sucrose	Δ*amyR*Δ*inuR* sucrose	Control inulin	Δ*amyR* inulin	Δ*inuR* inulin	Δ*amyR*Δ*inuR* inulin	Δ*amyR* sucrose	Δ*inuR* sucrose	Δ*amyR*Δ*inuR* sucrose	Δ*amyR* inulin	Δ*inuR* inulin	Δ*amyR*Δ*inuR* inulin
NRRL3_7700	*agdA*	68.855	6.861	302.128	15.133	134.737	12.070	23.460	11.342	**0.098**	**3.813**	**0.203**	**0.072**	**0.140**	**0.076**
NRRL3_2524	*agdB*	11.653	4.330	104.419	204.384	70.239	34.984	304.138	265.295	0.337	**7.397**	**14.870**	0.394	**3.472**	**3.195**
